# Increase in body size is correlated to warmer winters in a passerine bird as inferred from time series data

**DOI:** 10.1002/ece3.1323

**Published:** 2014-12-05

**Authors:** Mats Björklund, Antoni Borras, Josep Cabrera, Juan Carlos Senar

**Affiliations:** 1 Department of Animal Ecology, Evolutionary Biology Centre, Uppsala UniversityUppsala, Sweden; 2 Evolutionary and Behavioural Ecology Associate Research Unit, CSIC, Natural History Museum of BarcelonaBarcelona, Spain

**Keywords:** Citril finch, climate change, phenotypic evolution, plasticity, selection, time series

## Abstract

Climate change is expected to affect natural populations in many ways. One way of getting an understanding of the effects of a changing climate is to analyze time series of natural populations. Therefore, we analyzed time series of 25 and 20 years, respectively, in two populations of the citril finch (*Carduelis citrinella*) to understand the background of a dramatic increase in wing length in this species over this period, ranging between 1.3 and 2.9 phenotypic standard deviations. We found that the increase in wing length is closely correlated to warmer winters and in one case to rain in relation to temperature in the summer. In order to understand the process of change, we implemented seven simulation models, ranging from two nonadaptive models (drift and sampling), and five adaptive models with selection and/or phenotypic plasticity involved and tested these models against the time series of males and females from the two population separately. The nonadaptive models were rejected in each case, but the results were mixed when it comes to the adaptive models. The difference in fit of the models was sometimes not significant indicating that the models were not different enough. In conclusion, the dramatic change in mean wing length can best be explained as an adaptive response to a changing climate.

## Introduction

It is well established that the climate is changing with increasing temperatures as one of the factors that is changing. How animal populations respond to this is, however, a matter of debate. Given that the change in temperature may lead to selection, and the fact that most traits host measurable amounts of additive genetic variation, we would expect that populations respond to selection in an adaptive way, that is, by a genetic change due to selection. However, it has been remarkably difficult to show this in natural populations (e.g., Charmantier and Gienapp 2014), and one of the more remarkable phenotypic changes over time, breeding phenology in great tits, has been shown to be due to plasticity alone (Charmantier et al. 2008). A plastic response can be adaptive as well, but will not lead to a persistent change in the population. A great deal of effort has therefore been devoted to test whether a change is plastic or due to a genetic change as a result of selection (Merilä and Hendry 2014), and plasticity has been used as a null model against which genetic changes are tested. As pointed out by Merilä and Hendry (2014), plasticity is not well suited as a null model because this is a causal hypothesis itself, and if null models should be used, genetic drift models are more appropriate. Thus, a dichotomization of either a genetic or a plastic change is not biologically realistic because in most cases, we have both factors acting at the same time to a varying relative extent. This is, for example, clearly expressed in the Price theorem where change between generations in traits means is the sum of selection and “transmission bias”, that is, factors such as plasticity (e.g., Rice 2004).

Long-term trends in mean phenotypes are well known, and considerable theoretical effort has been devoted to understand the maximum rate of change over longer time periods, given the amount of additive genetic variation and strength of selection (reviewed in Kopp and Matuszewski 2013). To be able to know the potential and extent of genetic change as a response to changing environmental conditions, we need to have knowledge of the amount of additive genetic variation both before the trend starts and in the end, data that are rarely known. Merilä and Hendry (2014) summarize a number of approaches to get hold of this important information, but we have to face the fact that for the vast majority of natural populations we will not be able to get this data due to all sorts of reasons, logistic not the least. Thus, other means to understand the background of long-term phenotypic changes are necessary.

**Figure 1:**
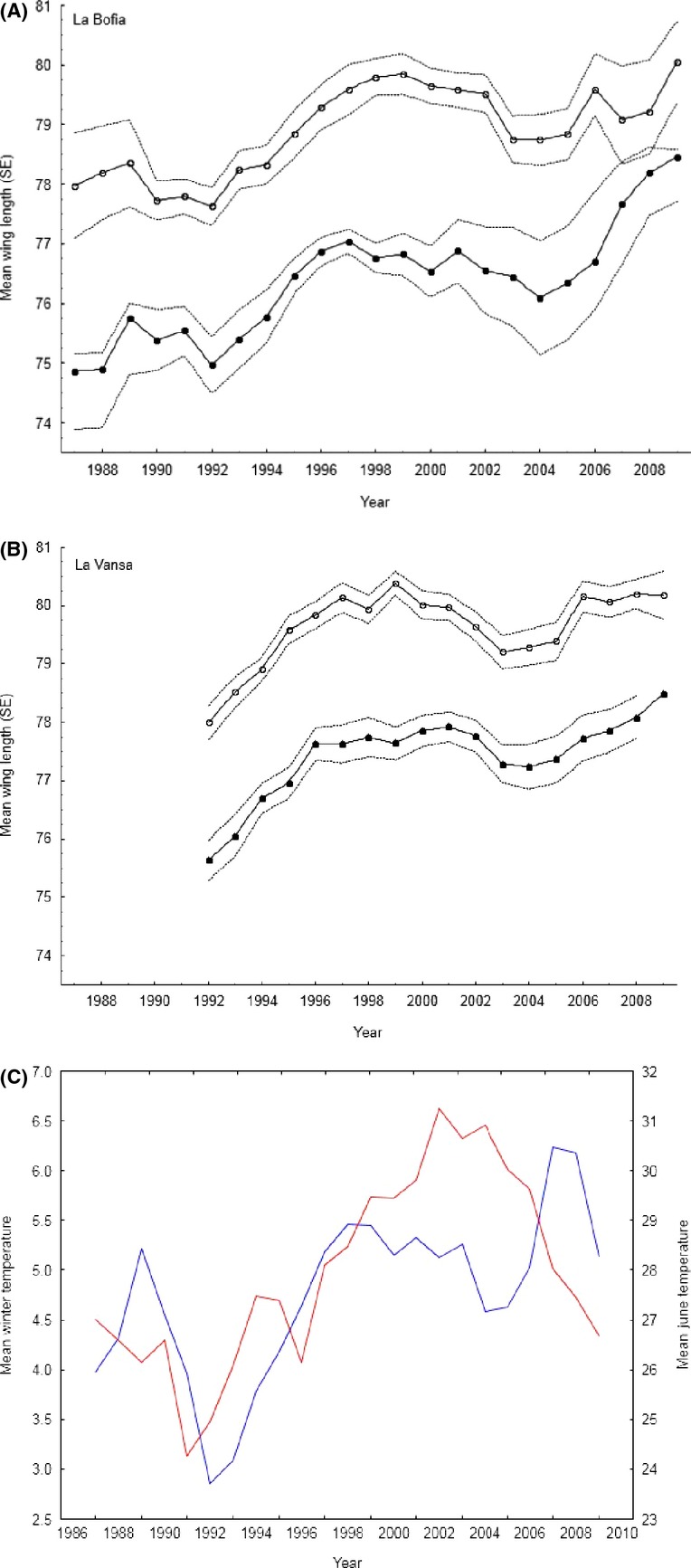
Mean wing length (±SE, dotted lines) over time for (A) males (open circles) and females (filled circles) in La Bofia, (B) the same for La Vansa, (C) mean winter temperature (blue line) and mean temperature in June (red line). There is a positive trend in mean winter temperature over time (*b *=* *0.069, *F*_1,23_ = 47.4, *P *=* *0.04), but not in summer temperature (*b *=* *0.12, *F*_1,23_ = 2.77, *P *=* *0.11). Time series smoothed by a 3-point moving average.

A complicating factor when studying possible effects of global warming on natural populations is that the effects can vary spatially depending on the overall ecological conditions for a certain population, and long-term studies on multiple populations are necessary for this. The citril finch (*Carduelis citrinella*) is an ideal model species for such a study. The species inhabits the high mountains of southwestern Europe, with higher densities being present in the Pyrenees mountain range (Borras and Senar 2003). Previous studies have shown that different slopes of these mountains, especially in the eastern range, can differ markedly in environmental characteristics. The species displays a marked local differentiation to this habitat heterogeneity, showing significant differences in wing length and genetically between localities (Senar et al. 2006). The mean wing length has increased substantially over time in the two populations (Fig.1A and B), such that females now have the same size as the males had in the late 1980's and early 1990's. The mean winter temperature has increased steadily from 1986 to 2010 (Fig.1C), but this is not the case for summer temperature (Fig.1C). In accordance with these observations, we predict that if a changing climate affects the population of citril finches, then it is correlated to the changing winter temperatures, and not the change in summer temperature. Furthermore, we predict that the extent of change in wing length is correlated to the magnitude of change in winter, but not summer temperature. We find that change in mean wing length is correlated with changes in winter temperature.

## Material and Methods

### Study populations and field methods

Citril finches were captured at two breeding sites approximately 5 km apart, in the Pre-Pyrenees, approximately 100 km northwest of Barcelona (NE Spain). Both sites are at approximately 2000 m elevation and are on opposite slopes of a 2378 m mountain (Port del Comte): La Bofia (41°10′N 1°32′E) faces south and its habitat intermixes Mountain Pines *Pinus uncinata* with subalpine meadows; La Vansa (41°12′N 1°35′E) faces north and its habitat is dominated by open Mountain Pine forests. The difference between the two localities is more extensively described in Senar et al. (2002, 2006). Due to the differences in habitat, the populations differ in a number of ways such that La Vansa birds enjoy higher survival rate, a higher breeding success and molt at a higher speed (Senar et al. 2002; Borras et al. 2004; Förschler et al. 2005). The populations move down in the nearby valleys over winter.

Birds were ringed from 1986 to 2010 in La Bofia and from 1991 to 2010 in La Vansa with most (approx. 99%) capture occurring between 1 April and 30 October. We captured a total of 9365 citril finches over the years (Vansa 4992, Bofia 4373). Birds were captured with mist nets and marked with numbered aluminum rings on capture. Sex and age were determined according to Svensson (1992); we defined juveniles as hatching year birds (EURING 3J and 3) and adults as after hatching year (EURING 5 and 6). For the analysis of biometric differences, we differentiated yearlings (EURING 5) from true adults (EURING 6). In the analysis, we only used the adult birds from each locality resulting in 637 males and 318 females from La Bofia, and 1149 males and 598 females from La Vansa. We measured wing length from the bend of the wing to the tip of the longest primary feathers, using the method of the maximum chord (Svensson 1992). All basic data are given in Data S1.

### Evaluation of environmental covariates

Meteorological data were available from Can Cabot station, located in Riner (Solsones, Lleida). This is a typical citril finch wintering area, down of Port del Comte mountains (Borras et al. 2010) and very close to Bofia/Vansa area (26 km), and previous analyses in our study area showed that meteorological data from different stations were highly correlated (Senar et al. 2002). Data used are presented in Data S1.

To summarize the climate data, we made a principal components analysis (PCA) using the cross-correlations between climate parameters over time using the winter data and the summer data separately (Table1). To evaluate the number of significant components, we used the broken-stick model of Jackson (1993), where the expected eigenvalues in a random correlation matrix are calculated as

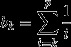
where *p* is the number of variables and *b*_*k*_ is the size of the *k*th component. Eigenvalues larger than the expected are significant and amendable for interpretation. Thus, the expected three-first eigenvalues in the winter data set with seven variables are 2.6, 1.6 and 1.09, and for the summer data set with six variables 2.45, 1.45 and 0.95. For the winter data, the first two eigenvalues were 4.02 and 1.69, and thus, we used the first two components (Table1). For the summer data set, the two-first eigenvalues were 4.31 and 0.69, and thus, only the first principal component is significant (Table1). Loadings were significant if they were larger than (# variables * # components) * 0.05 (Franklin et al. 1995), and thus for the winter data set, loadings larger than 0.7 were significant, and for the summer data set, the same figure was 0.3. Note that this applies only to the original scale of the eigenvectors and not the unit standardized eigenvectors that is normally displayed in a PCA. The values given here are standardized to unit length.

**Table 1:** Results from the principal components analysis using the climate variables, and the correlation between mean values in each sex and population at lag periods 0 (same year) and 1 (the year after) with the pc-scores. Bold figures are significant

(a) Winter		
Climate variable	PC1	PC2
Winter Temp	**0.34**	0.44
Mean Min temp	**0.49**	0.08
Minimum Temp	**0.36**	−0.07
No. freeze days	−**0.44**	−0.16
Extreme Temp	−**0.45**	−0.12
Winter rain (mm)	0.19	−**0.65**
Days of rain	0.29	−**0.58**
Eigenvalue	4.03	1.69
%	57.4	24.1
Lag 0		
Bofia males	−0.13	−0.04
Bofia females	**0.54**	−0.02
Vansa males	0.00	0.26
Vansa females	0.19	0.19
Lag 1		
Bofia males	**0.37**	0.07
Bofia females	0.16	−**0.35**
Vansa males	**0.50**	0.20
Vansa females	**0.45**	0.28

(b) Summer	
Climate variable	PC1
Max Temp June	−**0.45**
Mean Temp June	−**0.45**
Rain (mm)	**0.39**
Days of rain	**0.41**
Days over 30C	−**0.38**
Days over 35C	−**0.36**
Eigenvalue	4.31
%	71.8
Lag 0		
Bofia males	−0.01
Bofia females	**0.36**
Vansa males	−0.07
Vansa females	0.00
Lag 1	
Bofia males	−0.13
Bofia females	−0.08
Vansa males	−0.11
Vansa females	−0.30

In the winter data did PC1 account for 54.9% of the variance among years and PC2 25.6%. PC1 summarizes temperature with high values describing high temperatures, and PC2 describes temperature in relation to rainfall with high values represent warm and dry conditions. For the summer data, PC1 accounted for 72% of the variation and was the only significant component. PC1 describes rainfall in relation to temperature with high values represent wet and cold weather. We calculated the PC-scores for each year and made new cross-correlations between the finch data and the different PC's.

### Statistics

To test for a correlation of climate parameters and wing length, we used the cross-correlation of the time series (Chatfield 2004). This was performed in two ways, first, by the correlating climate variable at time *t* with the mean values at time *t*, that is, the same time period, here called lag 0. Secondly, we made the cross-correlation with the climate variable at time *t* with the mean value at time *t + 1*, that is, with 1 year of delay in the mean values, here called lag 1. To remove the possible effects of temporal autocorrelations, we prewhitened and detrended both time series by regressing the time series against time and used the residuals before calculating the cross-correlation to avoid spurious significance levels (c.f. Chatfield 2004). Test of significance of the cross-correlation was performed by a randomization procedure where new data sets were created with individuals drawn for each year from the pooled sample. The mean values from each year then constituted a new time series of wing lengths, which then was treated the same way as the original data (see above). We ran this 10,000 times and the proportion of times we found as large or larger cross-correlations as the one observed is then the *P*-value.

### Testing models of change

There are many possible causes for a trend like the ones found here. We will test seven different general models, two nonadaptive and five adaptive models either incorporating plasticity or selection or both. Details of each model are given below. The general approach was as follows. We wanted to test which model is the most likely one to have generated the data by generating distributions using these models. In each case, we ran the models 50,000 times to get the distributions. We evaluated the models by two criteria: the probability of getting a cross-correlation as large, or larger, as the one observed, *P*(*r*), and the probability that the time series ends with a mean value similar to that observed, *P*(*x*). *P*(*r*) was estimated by counting the number of times we found a cross-correlation as large, or larger, as the one observed. This is the probability of getting the cross-correlation given the model, *P*(*r*∣M), or *P*(*r*) for short. *P*(*x*) was estimated by counting the number of times a simulated time series ended with a mean value within one standard error from the observed mean value. This gives the probability of getting the mean value given the model, *P*(*x*∣M), or *P*(*x*) for short.

We evaluated the different models using a Bayesian approach. This can be performed by noting that *P*(*x*∣M) ≡ *L*(M∣*x*), that is, the likelihood of the model given the data (e.g., Congdon 2001). We already have the likelihood of obtaining a certain cross-correlation given the model, *L*(M∣*r*) = *P*(*r*∣M). Now, we can use the probability of getting the observed mean given the model (*P*(*x*∣M), as a prior as we know that the population actually got there, and thus weigh the likelihood by the prior to get the posterior probability of getting a certain cross-correlation, divided by the probability of the data as given by Bayes theorem. We then calculated the Bayes factor by relating the model with highest posterior probability to the other *i* models (*K* = *P*_max_/*P*_*i*_). For simplicity, we display the Bayes factor as 2Ln (*K*). For a very detailed summary of the procedure, we refer to Congdon (2001, p. 465–480).

In addition to comparing the fit of the models to the observed cross-correlation between the environmental factors and the mean values, we also compared the fit between the simulated time series and the observed one. This was performed by comparing the simulated mean (*x*_sim_) to the observed mean (*x*_obs_) each year and calculating the probability that the simulated mean was equal to the observed one by calculating a standard deviate = (*x*_sim_ − *x*_obs_)^2^/SD_obs_ and then calculating the corresponding probability using the standard normal curve. Thus, for each year, we obtained a probability that the simulated mean value is similar to the observed one. We then combined the yearly probabilities, Σln(*P*_*i*_), and thus obtained the likelihood of the data given the model. We used the same prior as above and calculated Bayes factors accordingly. By doing this, we get a distribution of likelihoods and we compare the models by looking at the mean of the distribution (*L*_mean_) and by the highest likelihood (*L*_max_; i.e., the smallest log-likelihood value). Models were then compared by means of Bayes factors as above.

Some of the parameters involved are the same for all models. Heritability, *h*^*2*^, could not be estimated directly using the real data but were taken from a normal distribution with mean 0.5 and a standard deviation of 0.1. This results in 95% of the values ranging between 0.3 and 0.7. This corresponds to values of morphological traits in other natural populations (Mousseau and Roff 1987). A new value of heritability was drawn for each run.

Another parameter is effective population size, *N*_e_. We have estimates of *N*_e_*μ*, where *μ* is the mutation rate, from each of the populations, and for Vansa, this equals 0.224 and for Bofia 0.305 (Senar et al. 2006). To get an estimate of *N*_e_, we used values of *μ* ranging between 0.0001 and 0.005 (drawn from a uniform distribution), which seems to be realistic for microsatellites (Li et al. 2002). We used a new value of *μ* for each run. The simulated values of *N*_e_ had medians of 122 (95%: 63–1358) and 90 (95%: 46–948) in La Bofia and La Vansa, respectively.

#### Model 1 sampling

The first of our four models assumes that the results we got are a product of sampling alone. Thus, as we have a limited sample, the cross-correlation might be a result of chance alone, that is, the mean has not changed over time and the cross-correlation is a spurious result of sampling. We simulated this by combining data from all years and for each year (*i*) randomly sampled *N*_i_ individuals, the same number as in the real data set. We then calculated the mean values for each year and made the cross-correlation between the different climate variables and the simulated time series of means as above.

#### Model 2 drift

It is well known from any standard textbook in population genetics that genetic drift can generate trends over time, even if the expectation of change is zero every generation. Thus, the trends we observe can be an effect of genetic drift alone. Genetic drift can be modeled as a normal deviate with zero expectation and a variance of 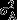/*N*_e_, that is, the additive genetic variance divided by effective population size (Falconer and Mackay 1996). We do not have empirical estimates of 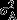, and thus, we used the fact that 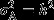 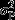, where *h*^2^ is the heritability and 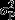 the phenotypic variance, which we do have information of Falconer and Mackay (1996). We started with the mean value for the first year in each population and sex and then added a value drawn of drift each generation;

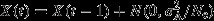


#### Model 3 plasticity

An obvious possible adaptive explanation is phenotypic plasticity (e.g., Merilä and Hendry 2014; Valladeres et al. 2014). However, this is notoriously difficult to model as there are a number of factors that we need to know from each population and this kind of data is rarely available for any population (Merilä and Hendry 2014). Thus, we use a simple model based on the data that is available acknowledging that the real situation might be far more complex. However, in the absence of data, a simple model is more parsimonious. Thus, our main hypothesis is that the mean values have not changed genetically but stayed the same over years, and the trend is a result of a plastic response to the environment that has changed over time with a different slope and intercept of plasticity for each individual. We have data on the same individuals from different years, and hence, we can estimate the amount of change in wing length in relation to a change in a climate parameter (Charmantier et al. 2008) and use this as a crude measure of plasticity. We found that wing length in 1 year was related to the amount of rainfall during the molt the year before (summer). Thus, we used the following relation

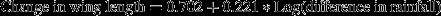


This model was significant (*F*_1,60_ = 9.09, *P* = 0.0038, *R*^2^ = 0.13), as was the intercept (*P* = 0.000025) and the slope (*P* = 0.0038). Each year, we sampled the same number of individuals as in the original sample, calculated the difference in rainfall between year *x* and *x*−1 and calculated the new phenotype according to the regression model above. To simulate individual variation in response to the different amounts of rainfall, we used the variance of the slope and the intercept and created new individual intercepts and slopes with values drawn from an *N* (0.702, 0.221) and *N* (0.221, 0.073) distribution, respectively. Thus, 95% of the intercepts lie between 0.4 and 1.0, and 95% of the slopes lie between 0.079 and 0.37. Not all individuals change between years, thus for each individual, there is a probability of change (PP) with turned out to be 0.75 (same for all groups) with a confidence interval of 0.64–0.84. Thus, for each run, we first calculated a probability that an individual had a plastic change using a normal approximation to the binomial with a mean of *N*_i_ PP and variance *N*_i_ PP (1−PP). This gives the number of individuals that are plastic so this was transformed to a proportion by dividing by *N*_i_. Thus, the model of plasticity is

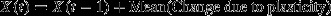


#### Model 4 constant selection

The simplest model of selection is where we assume that there is a new adaptive peak at the last year (*T*) of study and that there has been constant selection from year 1 to the last year. Thus, the selection differential (s) is (*X*[*T*] – *X*[1])/*L*, where *L* is the number of years. The response to selection every generation was calculated using the Breeder's equation, Δ*z* = *h*^2^s, where *h*^2^ is heritability as defined above. We also incorporated a change due to drift each generation. The model of change is thus

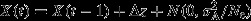


#### Model 5 fixed optimum weak selection

This model is similar to the preceding one except that we assume that there is stabilizing selection acting at the optimum and that selection change each year as a function of the distance to the new adaptive peak. Thus, the selection acting each year is

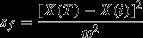
where *ω*^2^ is the strength of stabilizing selection. We used a fixed value of 20 to mimic weak stabilizing selection (to be relaxed below). The response to selection is thus, *Δz*_*f*_* = h*^*2*^*s*_*f*_. We also incorporated drift in this model and thus the model of change is as above but with the selection calculated differently.

#### Model 6 fixed optimum weak selection with plasticity

In this model, we combine Model 3 and Model 5 and thus the model of change is

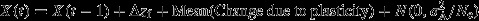


#### Model 7 moving optimum

In this model, we assume that the optimum changes every year (*i*) and is strictly related to the climate parameters. This was implemented as

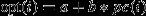
where pc(*i*) is the pc-score each year (see above). The slope (*b*) and the intercept (*a*) were determined by taking the first and last values of the mean values (*y*) and regressing those on the first and last values of the pc-scores (*x*). This assumes that populations are at the adaptive peak at the first and last years of study. In this model, we also let *ω*^2^ vary between runs. Based on the data in Estes and Arnold (2007) and Kingsolver et al. (2001), the mean value of *ω*^2^ is 11. The distribution is clearly skewed and to model this we draw values of *ω*^2^ from a log-normal distribution with mean 2.4 (log[11]) and a standard deviation of 0.6. This creates values centered at around 11 and with a long right-hand tail (see Fig. 7 in Estes and Arnold 2007). Thus, we calculated selection as

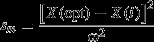


The response to selection is thus *Δz*_*m*_* = h*^*2*^*s*_*m*_*,* and the model of change over years is then

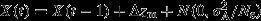


We note that there are an infinite number of different models that can be used, but we are here interested in testing a few, simple models that differ in important respects and will thus be able to reject certain factors based on the outcome of the model comparisons.

## Results

**Figure 2:**
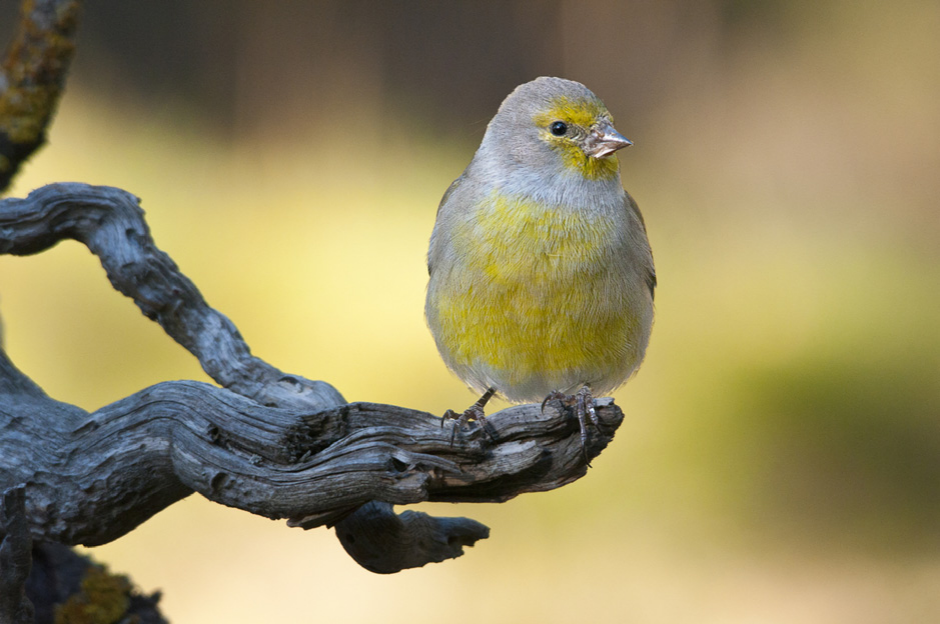
The study organism, the citril finch (*Carduelis citronella*).

La Bofia males changed 1.29 standard deviations (SD), La Bofia females changed 1.53 SD's, La Vansa males changed 1.57 SD's and La Vansa females changed 2.90 SD's (LR = 33.5, 24.2, 71.3, 30.1, respectively, *P *<* *0.001 in all cases, Fig.2). The rate of change measured in haldanes (phenotypic standard deviation/generation) was 0.080 (95% bootstrap interval = 0.065–0.094) and 0.12 (0.10–0.014) in La Bofia males and females, respectively, and 0.14 (0.12–0.16) and 0.26 (0.22–0.31) haldanes in La Vansa males and females, respectively.

**Figure 3:**
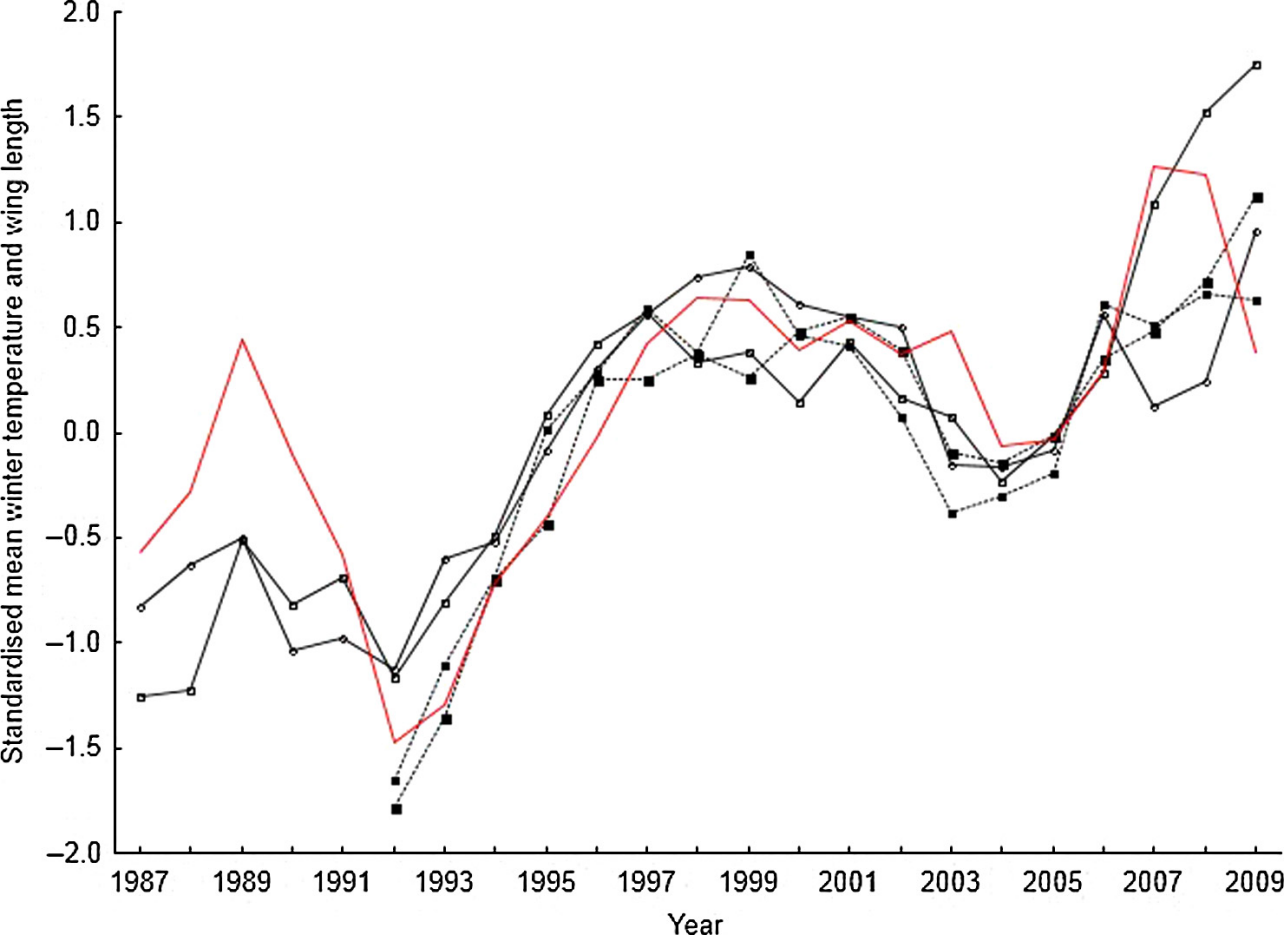
Standardized mean wing length and winter temperature (red line). Solid lines are male and females from La Bofia, and dotted lines males and females from La Vansa. Time series smoothed by a 3-point moving average.

Wing length in La Bofia females was positively correlated to winter PC1 in the same year (Table2), but in males from La Bofia and males and females from La Vansa, we found a positive correlation with winter PC1 and wing length the year after (Table1). This correlation means that the population means are higher after a warm winter, but with 1 year of delay. This is confirmed by looking at winter temperature alone where wing length was positively correlated to wing length (Fig.3). Wing length in La Bofia females was also positively correlated to increasing summer PC1 scores, that is, wet and cold summers increased the wing length of females. There was a significant cross-correlation between males and females in La Vansa (*r* = 0.71, *P* = 0.0012), but not in La Bofia (*r* = 0.36, *P* = 0.072). There was a significant cross-correlation between females in both populations (*r* = 0.56, *P* = 0.011) and males (*r* = 0.86, *P* = 0.000092).

**Table 2:** Result from the simulations. The columns show the group, the period, the model used in the simulations, the probability of obtaining a cross-correlation as high as that observed, the probability that the end point in the simulation is within 1 SE of the observed mean, the relative fit of the model given the data (*K*) expressed as the Bayes factor 2ln*K*, the best fit of a particular model to the data (*L*_max_) and relative fit expressed as the Bayes factor, the mean fit of a particular model to the data (*L*_mean_) and relative fit expressed as the Bayes factor. Bold figures indicate the best model

			Mean fit						
Group	Period	Model	*P*(*r*)	*P*(*x*)	Bayes	*L*_max_	Bayes	*L*_mean_	Bayes
Bofia M	Winter 1	Sampling	0.033	0	15.3	−253.1	498	−322.4	565
		Drift	0.031	0	15.4	−58.3	109	−129.6	179
		Plasticity	0.041	0	3.4	−12.1	5	−63.9	36
		Constant	0.022	0.06	**0**	−12.5	1.1	−59.5	23
		Optimal	0.003	0.15	2.0	−12.8	**0**	−48.8	**0**
		OptPlast	0.003	0.11	3.2	−16.1	7.3	−197.6	298
		Moving	0.002	0.22	2.5	−13.6	0.8	−82.3	66
Bofia F	Winter 0	Sampling	0.005	0.001	16.4	−15.6	33	−58.4	37
		Drift	0	0	31.9	−126.4	263	−157.5	243
		Plasticity	0.004	0.12	7.7	−8.9	10	−81.6	74
		Constant	0.13	0.16	**0**	−5.6	3	−98.7	107
		Optimal	0.002	0.22	8.0	−4.7	0.5	−48.9	7.1
		OptPlast	0.001	0.13	10.2	−5.0	2.1	−149.2	209
		Moving	0.001	0.29	0.8	−4.7	**0**	−45.6	**0**
Bofia F	Winter 1	Sampling	0.081	0	9.6	−13.9	30	−58.5	38
		Drift	0.031	0	19.4	−125.6	261	−157.4	244
		Plasticity	0.058	0.11	0.8	−9.3	11	−81.5	75
		Constant	0.032	0.16	1.3	−5.2	2.3	−98.8	108
		Optimal	0.039	0.23	0.2	−4.8	0.6	−49.0	8.1
		OptPlast	0.024	0.13	2.3	−5.4	3.0	−148.8	209
		Moving	0.035	0.29	**0**	−4.7	**0**	−45.2	**0**
Bofia F	Summer	Sampling	0.047	0.001	14.3	−17.4	37	−58.4	37
		Drift	0.089	0	20.9	−131.8	274	−157.4	243
		Plasticity	0.067	0.11	4.0	−11.1	15	−81.4	74
		Constant	0.12	0.16	2.3	−4.5	1.1	−98.5	107
		Optimal	0.17	0.36	**0**	−4.8	**0**	−49.0	6.5
		OptPlast	0.092	0.14	3.2	−5.3	3.0	−148.9	208
		Moving	0.15	0.28	0.7	−4.6	0.1	−45.5	**0**
Vansa M	Winter 1	Sampling	0.011	0.002	8.5	−5.8	16	−13	**0**
		Drift	0.012	0	17.8	−153.0	320	−229.5	443
		Plasticity	0.009	0.21	**0**	−2.3	**0**	−21.2	7.4
		Constant	0.011	0.033	3.3	−4.8	8.7	−106.3	181
		Optimal	0.010	0.035	3.2	−3.4	5.8	−42.0	53
		OptPlast	0.008	0.074	2.2	−2.8	3.0	−72.2	112
		Moving	0.006	0.14	1.7	−2.0	0.2	−25.1	16
Vansa F	Winter 1	Sampling	0.021	0	18.0	−7.4	6.6	−23.2	38
		Drift	0.023	0	18.0	−213.9	96	−247.5	487
		Plasticity	0.017	0.029	3.9	−1.6	1.0	−25.3	28
		Constant	0.025	0.024	3.4	−2.3	1.4	−87.9	153
		Optimal	0.028	0.042	2.1	−1.4	0.7	−14.7	6.0
		OptPlast	0.027	0.13	**0**	−1.3	0.2	−56.5	87
		Moving	0.015	0.17	0.6	−1.2	**0**	−13.1	**0**

**Figure 4:**
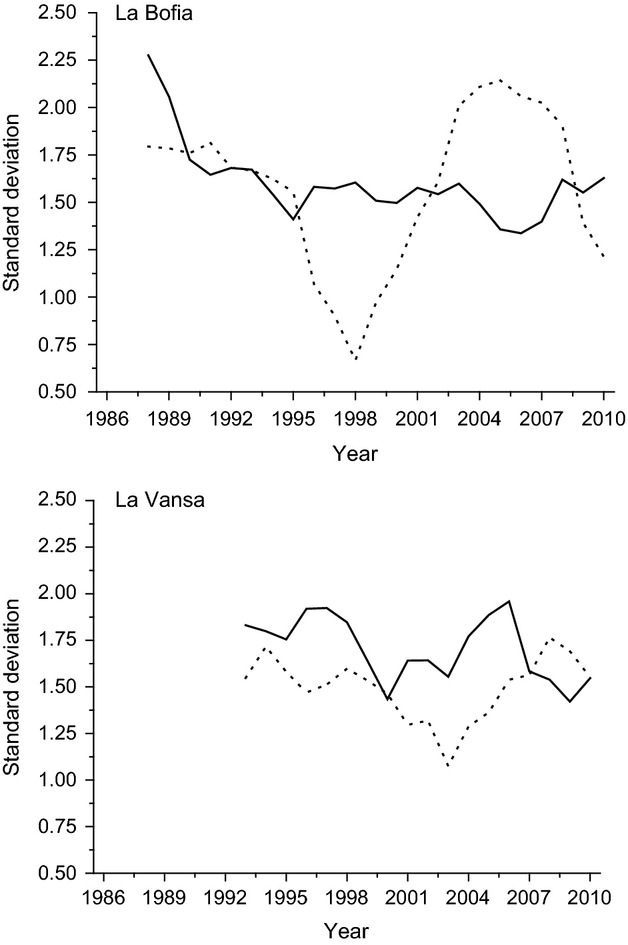
Phenotypic standard deviations over time. Solid lines males, dotted lines females.

Trait variances changed over years, but not in the way mean values changed (Fig.4). There were no correlations between the amount of change in mean values and change in variance in any of the groups (*r* = −0.12 and 0.11 for La Bofia males and females, respectively; *r* = 0.076 and 0.12 for La Vansa males and females, respectively).

**Figure 5:**
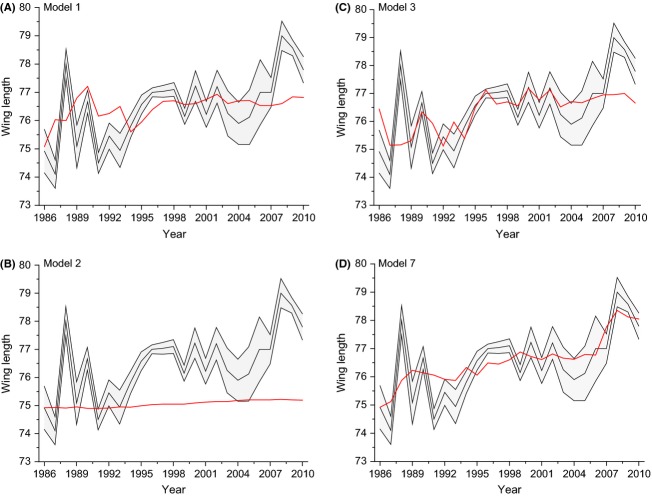
Examples of fit between data and four models using Bofia females as an example. The red line is the result of a simulation, and the black line the observed mean ± 1 SE (shaded area).

The comparison of possible models shows that the nonadaptive models could be clearly rejected when it comes to mimicking the correlation between the environmental variables and wing length. Examples of the simulations results are given in Figure5. The model of constant selection (Model 4) was the best model for males from La Bofia and females from La Bofia when it comes to winter period lag 0. The moving optimum model (Model 7) was the best model for females from La Bofia for the winter period at lag 1, while the model with a single optimum (Model 5) was the best for the summer period in La Bofia females. In La Vansa, the plasticity model (Model 3) was the best for males, and the combined optimum and plasticity model (Model 6) was the best one for females. However, as is evident from Table2, many models differed little in terms of posterior probabilities. If we concentrate on Bayes factors larger than 6, which indicates strong evidence of a difference in support, we find that none of the adaptive models are rejected for males from La Bofia, winter lag 1 and summer in females from La Bofia and for both males and females from La Vansa. Two models stand out for females from La Bofia in the winter period lag 0 and that is the constant selection model (Model 4) and the moving optimum model (Model 7), while the remaining models could be rejected.

Further information on the success of the models to simulate the patterns observed can be obtained by analyzing *P*(*r*) for the different models. In most cases was *P*(*r*) low with the exception of the summer data for females from La Bofia, in other words, the models did a relatively poor job of recreating the correlation between the climate parameter and the mean values.

If we instead look at the ability of the models to simulate the means themselves, without reference to the correlation with the climate variable, we find a much better fit. For example, all adaptive models had an *L*_max_ in the range of −12 to −2. To set this in perspective, if all simulated mean values are exactly 1 standard deviation from the observed mean, *L*_max_ would be −22.8 for La Vansa. A value of −1.2 then corresponds to 0.02 SD's from the observed mean, which means that the simulated data are very close to the observed mean values. In fact, in all cases but for males from La Bofia was the fit between the simulated adaptive models and the observed data very good. However, this is the best possible fit and a perhaps a better measure is the *L*_mean_. Using these figures, it is clear that some models are, on average, still did quite well in replicating the observed data. For example, *L*_mean_ for the moving optimum model for females from La Vansa was −13.1, which corresponds to a situation where, on average, the simulated mean is around 0.5 SD's from the observed mean. Values larger than around −75 indicate that the distance between the observed and simulated mean is, on average, larger than 2 SD's.

## Discussion

We found correlations between winter conditions in 1 year and wing length in the next year. In particular, warmer winters correlated with larger individuals, and as the winters have become warmer over the years of study, so has the citril finches. The pattern is not entirely consistent, though, with the females from La Bofia being the exception. In La Vansa and in males in La Bofia, the correlation is between temperature in year *t* and wing length in year *t* + 1. This means that the effect of the temperature in one winter is seen in the summer the year after. In females in La Bofia, however, this effect is seen the same year, which suggests that the warmer temperature has a direct effect on these females, rather than the indirect effect as in the other groups. In addition, these females are also affected by summer conditions, which is not the case in the other groups. In particular, summers that are rainy in relation to the temperature seems to select for larger females. Furthermore, there was a delayed effect of winter conditions in females from La Bofia such that dry winters select for larger females in the year after. The differences in response are seen in the lack of a significant correlation between male and female time series in La Bofia, but which is highly significant in La Vansa. Furthermore, the correlation between females in the two populations was lower than the correlation between males.

The results also suggest that birds from the two habitats differ such the impact of changing winter temperature is stronger in La Bofia, which is probably an inferior habitat to La Vansa (Senar et al. 2002; Borras et al. 2004; Förschler et al. 2005). It needs to be stressed in this context that the birds overwinter in sympatry (Borras et al. 2010, 2011) and thus affected by the same winter conditions and that the differences in change between the years are due to an interaction between winter conditions and conditions the rest of the year that might differ between the two populations. Thus, the impact of a change in climate seems to be conditional on the quality of the habitats the populations are living in part of the year. This in turn strongly suggests that studies of effects of a changing environment should incorporate several populations in order to make more robust inferences. It needs to be remembered at this stage that what we have found is a strong correlation between changes in a climate variable and changes in mean phenotype, and not a causal relationship, and there might be another underlying factor behind this correlation.

**Figure 6:**
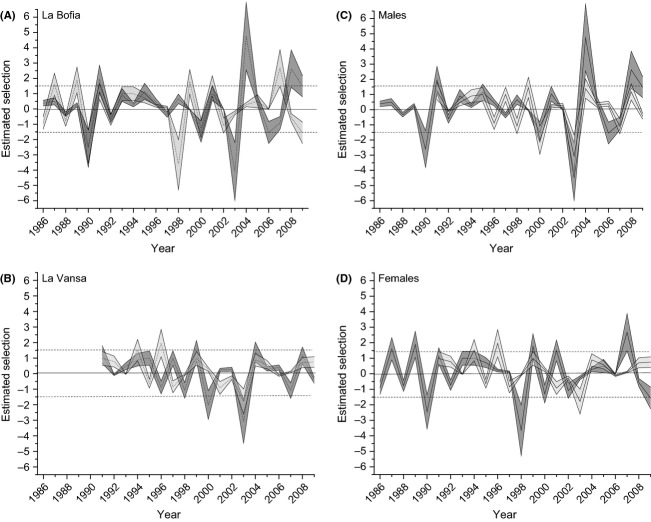
Estimated selection for each. The bands are the 95% intervals. The dotted lines represent the upper limit of observed selection coefficients taken from literature data. (A) Males and females from La Bofia, (B) males and females from La Vansa, (C) males from both populations, (D) females from both populations.

To get an understanding of the magnitude of selection that might have been acting, and if these values are reasonable we treated the difference between means in adult wing length as the response to selection (*R*). This means that the selection coefficient (*s*) can be estimated from the Breeder's equation as *s* = *R*/*h*^2^, where *h*^2^ is the heritability that can be estimated as above. The results from this rough estimate of selection can be seen in Figure6. To compare to other published estimates, the estimated selection is expressed in units of standard deviations. Basically all published estimates are within ±1.5 SD's, which corresponds to a truncation selection of the 15% largest (smallest) individuals, and this is denoted in Figure6 by the dotted lines. From this rough analysis it is clear that in almost all years is the estimated selection well within the magnitude found in other studies. The main exceptions are 2003 and 2004 in males from La Bofia, 2003 for males in La Vansa and 1998 in females from La Vansa, where the estimated selection is far from what is found elsewhere, that is, larger than 3 SD's. Selection intensities in that order correspond to truncation selection of the largest (smallest) 0.4%. If the breeding population is 2000 individual, this corresponds to 8 individuals breeding that year. This is obviously unrealistic, and hence, these values are composed of other factors as well.

One thing that needs to be remembered in these crude calculations is that there are several possible selection episodes between adult to adult change in mean, such as selection during breeding, selection of fledging individuals and selection on overwinter first-year birds. This means that the estimated selection is the composite of a number of selection episodes and thus, if they act in the same direction, easily can add up to larger numbers. For example, an estimated selection of 3 SD's might be a sum of four different episodes of 0.75 SD's. This is still intensive selection, but within the range of what has been seen in other populations (Kingsolver et al. 2001). Thus, the values obtained are perhaps not that extreme after all, even though without knowledge of the details of selection at other episodes, we cannot make stronger inferences.

One issue that complicates studies like this is the extent of phenotypic plasticity. To be able to understand the impact of plasticity, we need to know the reaction norm of each genotype, data that are basically impossible to get in a wild-ranging population like this, and this is a problem that is obvious in most natural populations. However, the crude measure of plasticity we used here, which is based on observed between-year changes in wing length, turned out to be the best model in one case (males in La Vansa) and with reasonable support in the other cases. In females from La Vansa, the model that combined plasticity and the new optimum had highest support. Thus, even if the mechanism of plastic response probably is more complicated than we have envisaged, the main conclusion is that plasticity quite likely has had an impact on the changes over years in wing length in the citril finch.

Long-term directional selection is expected to erode genetic variance, and if the genotype–phenotype map remains intact (i.e., constant heritability), we would expect this to been seen also in the phenotypic variance (Falconer and Mackay 1996) as a trend of decreasing phenotypic variance over time. However, we did not see any trend in decreasing variance in either sex or population (Fig.4). Thus, despite the substantial change in mean values, the phenotypic variation has been kept intact. Unfortunately, we have no information on the amount of additive genetic variation in this species, and it is basically impossible to obtain for logistic reasons. If genetic variation has decreased, but the phenotypic variance remains intact this must mean that heritability has decreased over time, or in other words, the organisms increased their level of plasticity over time. However, recent results from other study systems suggest that genetic variance can actually increase as a result of a changing environment through the expression of cryptic genetic variance (e.g., Paaby and Rockman 2014). The results suggest that selection has been weak enough to keep, at least, the phenotypic variance relatively stable over time.

The models we have used are admittedly simple, and there is an infinite number of ways to model processes that might match the observed trend. For example, selection on correlated traits and genetic correlations between traits within and between sexes has not been incorporated. We wanted to keep things simple using a minimum of unknown variables and still we were able to get a good match between the observed and simulated data. We could also exclude two nonadaptive models and concentrate on the adaptive ones. Often a dichotomy between plasticity and selection is made (reviewed in Merilä and Hendry 2014), but this is an unnecessary division of processes. A change in mean values between years could be due to either process or both in combination. Likewise, plasticity is to some unknown extent adaptive and amendable for selection as any trait. We know that there is plasticity in wing length in this species as about three-fourth of the individuals change their wing length between years. Wing feathers are molted every year, and hence, there is thus ample opportunity for local environmental and intrinsic physiological factors to interact, but we have so far no idea on the exact details on how this might work. We did find a relationship between change in wing length and rainfall during summer, which gives a hint on how plasticity works in this species.

The results presented here have strong implications for the study of climatic effects on natural populations. First, a sufficiently long time series is needed to capture the changes that might have occurred. If the time series is too short, then the changes in climate during the period studied might be too weak to result in any evolutionary change. This can be seen in Figures1, 3 where the winter temperature did not change much over a decade (i.e., 1997–2007), and there were no changes in mean wing length either. Second, populations even very close to each other might respond in different ways depending on local factors. Thus, the global impact might be enhanced in some populations and mitigated in other populations. This means that multiple populations need to be studied in order to control for local, population-specific factors. Third, changes in mean values as a response to a changing environment can be very fast on an evolutionary time scale. The changes observed here are large, but still within the range of those observed in other taxa (Kinnison and Hendry 2001), albeit in the upper range of the distribution. As an extreme case, Grant and Grant (1995; recalculated by Hendry and Kinnison 1999) found changes up 0.7 haldanes over two generations in Darwin's finches, although most rates of change are in the order of 0.03 (Kinnison and Hendry 2001).

In conclusion, the large change in mean wing length over 20–25 years in two populations of the citril finch is best explained by adaptive factors, such as selection and plasticity, alone or in some combination. The results also show that a change in an environmental factor, such as winter temperature, can lead to drastic changes in men phenotypes over very short time periods.

## Supporting Information

**Data S1.** Means, standard deviation and sample size for each year and sex for the two studied populations. The figures to the righthand-side of the Table are the meteorological data used in the analyses.
